# Role of income and government responsiveness in reducing the death toll from floods in Indian states

**DOI:** 10.1038/s41598-022-21334-w

**Published:** 2022-10-10

**Authors:** Yashobanta Parida, Joyita Roy Chowdhury, Swati Saini, Devi Prasad Dash

**Affiliations:** 1 Department of Economics, FLAME University, Pune, India; 2 Department of Economics, Delhi School of Economics, University of Delhi, New Delhi, India; 3 School of Management and Entrepreneurship - IIT Jodhpur, Jodhpur, India

**Keywords:** Environmental social sciences, Natural hazards

## Abstract

Floods are the most commonly occurring natural disasters in India due to India’s unique geographical location and socioeconomic conditions. Frequent flooding causes enormous loss of human lives and damage crops and public utilities. Furthermore, floods adversely affect economic development and increase the government's financial burden by increasing spending on various disaster mitigation measures. Recent empirical literature based on cross-national comparisons shows that disaster fatalities and damages are monotonically decreasing in per capita income. We challenge this view on the monotonic negative relationship between income and flood damages. We examine the non-monotonic (inverted U-shaped) relationship between per capita income and flood impact in terms of deaths, people affected, and damages due to floods in 19 major Indian states from 1980 to 2011, using Poisson and Tobit estimation methods. In particular, deaths and the population affected by floods increase with a turning point of income up to 882 US$ and 578 US$, respectively, and diminishes thereafter. Our results confirm an inverted U-shaped relationship between income and fatalities and the population affected by floods. In addition to income, we argue that government responsiveness plays an essential role in mitigating the risk of floods. We employ the fixed-effect Poisson estimation method to examine the government's role in protecting people against disaster risk, focusing on regional differences in India. Deaths from floods remain non-linear and follow the inverted U-pattern with respect to government responsiveness. However, the effect of government responsiveness on flood fatalities and flood damages is statistically insignificant. Our results further suggest that high-income states experience a lower death toll from floods. The high-income (rich) states are capable of incurring a higher threshold level of income and higher natural calamity expenditure to reduce flood fatalities and protect the population affected by floods than the low-income (poor) states. The poor states have minimal resources and face severe financial constraints to reduce the death toll from floods. From the perspective of public policy, the poor states, in particular, require an increase in income, better governance, and effective disaster management policies to mitigate flood impact.

## Introduction

India is one of the most flood-affected nations globally after China (Emergency Events Database, (EM-DAT))^1^. India’s geo-climatic conditions have caused severe flood fatalities and damages. Moreover, other factors such as higher flood-prone regions, irregular rainfall, higher population density, deforestation, illegal constructions, and environmental degradation have contributed to increasing flood damage in the Indian states. According to the EM-DAT, 194 floods occurred in China, which is the highest among the nations, followed by India (190), Indonesia (126), Philippines (109), and Bangladesh (69) over the period 1980 to 2011. Overall, in India, floods account for 53%, followed by cyclones (21%), landslides (10%), cold waves (6.4%), earthquakes (4.2%), and droughts (2%) over the same period. Flood is recorded as the costliest disaster in India, accounting for 68% of economic losses over all-natural disasters damages. It is India's second most lethal disaster after the earthquake. Between 1980 and 2011, floods were the costliest among all disasters in India, accounting for 68% of total disaster-related damages, followed by cyclones (16%), earthquakes (10%), droughts (4%), hear-wave (0.8%), cold-wave (0.29%) and landslides (0.11%). Moreover, earthquake is the most lethal disaster in India, accounting for 39 percent of deaths considering all-natural disaster deaths, followed by floods (32%), Cyclones (17%), heat-wave (6%), cold-wave (4%), landslides (2%), and droughts (03%).

India lost around 0.46% of gross domestic product (GDP), damages to crops stood at 0.18% of GDP, 1.6 million houses were damaged, damage to public utilities stood at around 0.21% of GDP, and 6% of the rural population were affected due to floods annually^2^. In addition, flood impact builds fiscal pressure on the government through spending on flood protection measures and creating flood-resilient infrastructure. Based on the socioeconomic condition of the states, high-income (rich) states in India have experienced lower flood fatalities, and fewer people have been adversely affected than the low-income (poor) states. Moreover, rich states spend more on post-disaster mitigation measures than poor states (See Supplementary Information, Appendix Table A.1). Many empirical studies show that countries with higher per capita income experience lower disaster deaths than developing countries^3,4^. We argue that per capita income is not adequate to minimize the disaster impacts. Therefore, the government’s role is also crucial in protecting citizens against natural disasters. In this context, few studies show that stronger institutions and improved governance help reduce deaths from natural disasters^3,5^. The Indian government spends around 0.23% of Gross State Domestic Product (GSDP) on relief distribution, evacuation, and rehabilitation measures (see Appendix Table A.1). Still, this proportion of public expenditure directed toward disaster risk reduction is not adequate for India. We argue that in addition to increasing per capita income level, the extent of government intervention should be enhanced to implement better disaster management efforts to mitigate the disaster risk in Indian states.

First time in the empirical literature^6^, analyzed the non-linear (inverted U-shaped) relationship between per capita income and various pollution indicators. Subsequently, in the economics of disaster literature, a few studies have empirically shown a non-linear (inverted U-shaped) relationship between disaster risk and income using cross-country panel data^7–9^. These studies show that initially, disaster risk increases with income and then declines when income has reached a certain threshold level. In the Indian context, no empirical study has addressed the inverted U-type relationship between flood fatalities and per capita income. We argue that flood fatalities and damages initially increase with increasing per capita income, and then deaths and damages decline when per capita income increases further. In a developing economy like India, societies spend more income on basic needs in the initial phase, and less money is spent on safety and disaster preparedness measures. Over the period, when society’s per capita income increases, the demand for safety rises because income provides insurance against natural disasters.

Similarly, we examine the inverted U-shaped relationship between government responsiveness and flood fatalities. The primary reason is that the government spends more on social security programs such as poverty reduction, health, and education in the initial phase. Therefore, fewer resources are devoted to disaster management efforts. This is consistent with the idea that individuals tend to spend more on basic consumption needs in the initial period and spend less income on disaster preparedness activities. Over the period, society’s demand for disaster mitigation measures increases, and therefore, eventually, the government budgetary allocation increases towards disaster risk reduction measures. Given that disaster preparedness and responses depend on the nation’s wealth and governance structure, we focus on two essential components: economic development (proxied by per capita income) and better governance (proxied by natural calamity expenditure) that can help mitigate damages and fatalities from natural disasters.

In this framework, our paper examines the impact of income and government responsiveness on fatalities and damages caused by floods in Indian states. Using a state-wise panel data on annual deaths and population affected due to floods in 19 Indian states from 1980 to 2011, our study has the following objectives: First, do income and flood fatalities (and population affected) exhibit a non-linear (inverted U-type) relationship in all Indian states? Second, when flood disasters occur, how many fewer deaths are caused in high-income (rich) Indian states than in low-income (poor) states. Third, does government responsiveness (proxied by natural calamity expenditure) reduce the flood impact (measured by deaths and population affected due to floods)? Government responsiveness is described as how the government responds during and in post-flooding situations and provides necessary services to citizens to minimize the flooding impact. This also includes ex-ante measures such as building up flood-resilient infrastructure and devising suitable flood control measures to alleviate flood risk.

We employ a fixed effect (FE) Poisson and FE negative binomial methods to achieve the aforementioned objectives. To analyze regional heterogeneity, we divide the Indian states into high-income (rich) and low-income (poor) states, following^10^. The low-income states include states located in India's north and eastern parts. All states are not equally affected by floods and are also not capable of spending substantially on natural disasters due to their locational disadvantages and lack of disaster-resilient infrastructure. For instance, the north-east and east states are highly vulnerable to floods and belong to the category of low-income states (see Appendix Table A.1).

We contribute to the literature by highlighting the non-monotonic relationship between per capita income and flood impact (measured by deaths and population affected by floods). We argue that flood fatalities and damages initially increase with an increase in income and then it declines when income rises further. Second, we argue that government responsiveness (proxied by natural calamity expenditure) also exhibits a non-linear relationship with deaths and damages caused by floods. The impact of per capita income and government responsiveness on flood fatalities is studied for India's high-income and low-income states while controlling socioeconomic factors, political alignment, and severity of floods. In the context of India, no empirical work exists that explains the non-linear relationship between income and deaths and damages caused by floods. Our focus on regional differences in India has much potential. In addition to income, institutions also play a critical role in protecting Indian states from disaster shocks. An empirical analysis of government responsiveness to mitigate flood disaster risk is missing in the literature. Therefore, we fill the gap by measuring the quantitative importance of government responsiveness to reducing flood impact.

## Subjects and methods

### Review of literature

Several empirical studies confirmed that higher Gross Domestic Product (GDP) per capita reduces disaster damages and fatalities^11–13^. These studies using macro panel data argued that countries with a higher GDP per capita experience lower deaths and damages from natural disasters. Parida^11^ employed instrumental variables (IV) Poisson and fixed effect (FE) Tobit estimation methods to examine the impact of economic development on flood fatalities and damages in Indian states. The results suggest that states with higher per capita income help reduce fatalities and damages. Anbarci et al.^12^ examined the impact of income inequality and per capita income on earthquake fatalities using cross country dataset. The FE negative binomial result shows that income inequality minimizes the earthquake fatalities. Strömberg^13^ examined the impact of per capita income and the role of governance on disaster fatalities using data from high, middle, and low-income countries. The ordinary least squares and instrumental variable (IV) estimates confirm that countries with better governance and higher income help minimize deaths from disasters. The study also shows that developed countries experience 70% lower deaths than low-income countries.

Toya and Skidmore^4^ emphasized the two essential components of the disaster-income-safety relationship. Using the ordinary least squares (OLS) estimation method, they showed that, on the one hand, a higher level of income raises the private demand for safety. On the other hand, high income enables individuals and countries to be better equipped to respond to disaster risks by employing several precautionary measures. Kahn^3^ used Zero-inflated negative binomial estimation to examine the impact of institutions’ quality and economic development on disaster fatalities. Their empirical findings show that fatalities reduce as income increases and that the number of deaths is higher in less democratic countries than in more democratic countries. Sadowski and Sutter^14^ examined the impact of income, occurrence of hurricanes, and higher population density on hurricane damages and fatalities in the United States. The Poisson regression estimates indicate that income is negatively related to damages while the occurrence of hurricanes is positively associated with fatalities.

Keefer et al.^15^ examined the relationship between earthquake propensity and earthquake fatalities using a zero-inflated negative binomial model. The empirical results confirm that mortality is lower in countries with higher earthquake propensity because of higher investment in earthquake preparedness. Cavallo et al.^16^ examined the economic losses due to the earthquake in Haiti. The fixed effects estimates confirm that higher earthquake mortalities lead to an increase in the damage caused by the earthquake in Haiti. Escaleras and Register^17^ employed a quantile regression method to analyze the relationship between fiscal decentralization and death from natural disasters using a cross-country dataset from 1972 to 2000. The results confirm that higher sub-national expenditure helps mitigate the death from disasters. In sum, all these studies suggest that fatalities and damages due to floods are monotonically decreasing in per capita income.

In contrast, another set of studies shows that the relationship between GDP per capita (or income per capita) and deaths and damages from natural disasters is not always monotonic; instead, it is non-monotonic that is characterized by an inverted U-shaped relationship, where damages from disasters initially increase and then finally decrease with income. Kellenberg and Mobarak^7^ examined the non-linear (inverted U-type) relation between income and deaths from disasters using negative binomial and pooled generalized least squares (GLS) methods. The results confirm that disaster deaths increase until a certain threshold level of GDP per capita is reached and then decline when the GDP per capita crosses the threshold level. Ferreira et al.^9^ evaluated the non-linear relation between economic development and flood fatalities using FE Poisson and FE negative binomial. The results show that income produces a significant indirect effect on flood fatalities and that this effect is non-monotonic, with net reductions in disaster deaths taking place in low-income countries. Schumacher and Strobl^8^ showed that the non-linear relationship between economic losses caused by disasters and economic development depends on how the particular countries are exposed to disasters. Using the Tobit estimation method, they empirically showed how countries with a low disaster risk face increasing losses in the initial stage, followed by a phase where they face decreasing losses with increasing economic development. Results suggest an inverted U-shaped relationship between economic development and disaster losses for the countries with low disaster risk. At the same time, countries experiencing a high risk of disasters suffer decreasing losses initially and then increasing ones with rising economic development.

Besides GDP per capita’s impact, countries with improved institutional quality experience a lower impact of deaths and lower economic damages from natural disasters^3^. Raschky^5^ analyzed the relationship between the institution's role and losses from natural disasters using cross country dataset. The OLS estimates show that better institutions (a proxy of government stability) that bring an improved allocation of resources help reduce disaster losses. North^18^ pointed out that countries with better institutions and governance structures invest more in physical and human capital and efficiently utilize their factor endowment. This shows a nation’s ability to mitigate the adverse impacts of natural disasters when equipped with better institutions and effective governance. Garrett and Sobel^19^ employed the Poisson regression model to analyze the relationship between disaster declaration and allocation of disaster expenditure in an election year in the United States. The empirical results confirm that disaster declarations are higher in states with political importance to the presidents. Besley and Burgess^20^ analyzed the political economy of government responsiveness using state-wise data in India. The FE estimates show that greater flood damages and higher newspaper circulation improve government responsiveness in the Indian states.

### Empirical methodology

We estimate three equations to analyze the non-linear (inverted U-type) relationship between per capita income and flood measures such as fatalities, the population affected, and damages due to floods in high-and low-income states of India.

1 $$ \begin{aligned} FF_{st} & = \beta_{0} + \beta_{1} ln{\text{PCY}}_{{{\text{st}} - 1}} + \beta_{2} lnPCY_{st - 1}^{2} + \beta_{3} \left( {\frac{NCE}{{GSDP}}} \right)_{st - 1} + \beta_{4} \left( {\frac{NCE}{{GSDP}}} \right)_{st - 1}^{2} + \beta_{5} {\text{SED}}_{{\text{st }}} + \beta_{6} {\text{NED}}_{{\text{st }}} \\ & \quad + \beta_{7} {\text{PAD}}_{{\text{st }}} + \beta_{8} {\text{ AD}}_{{\text{st }}} + \beta_{9} {\text{Z}}_{{{\text{st}} - 1{ }}} + \theta_{s} + \gamma_{t} + \mu_{1,st} \\ \end{aligned} $$

2 $$ \begin{aligned} PA_{st} & = \alpha_{0} + \alpha_{1} ln{\text{PCY}}_{{{\text{st}} - 1}} + \alpha_{2} lnPCY_{st - 1}^{2} + \alpha_{3} \left( {\frac{NCE}{{GSDP}}} \right)_{st - 1} + \alpha_{4} \left( {\frac{NCE}{{GSDP}}} \right)_{st - 1}^{2} + \alpha_{5} {\text{SED}}_{{\text{st }}}  \\ & \quad + \alpha_{6} {\text{NED}}_{{\text{st }}} + \alpha_{7} {\text{PAD}}_{{\text{st }}} + \alpha_{8} {\text{ AD}}_{{\text{st }}} + \alpha_{9} {\text{Z}}_{{{\text{st}} - 1{ }}} + \theta_{s} + \gamma_{t} + \mu_{2,st} \\ \end{aligned} $$

3 $$ \begin{aligned} FD_{st} & = \delta_{0} + \delta_{1} ln{\text{PCY}}_{{{\text{st}} - 1}} + \delta_{2} lnPCY_{st - 1}^{2} + \delta_{3} \left( {\frac{NCE}{{GSDP}}} \right)_{st - 1} + \delta_{4} \alpha_{4} \left( {\frac{NCE}{{GSDP}}} \right)_{st - 1}^{2} + \delta_{5} {\text{SED}}_{{\text{st }}} \\ & \quad + \delta_{6} {\text{NED}}_{{\text{st }}} + \delta_{7} {\text{PAD}}_{{\text{st }}} + \delta_{8} {\text{ AD}}_{{\text{st }}} + \delta_{9} {\text{Z}}_{{{\text{st}} - 1{ }}} + \theta_{s} + \gamma_{t} + \mu_{3,st} \\ \end{aligned} $$

where *s* represents State {1….19} and *t* represents the year {1980…2011}, $$FF_{st}$$ denote fatalities in state **s** and year **t**, $$PA_{st} $$ indicates population affected due to floods, and $$FD_{st }$$ denotes natural logarithm of the ratio of flood damage over Gross State Domestic Product (GSDP); $$ ln{\text{PCY}}_{{{\text{st}} - 1}}$$ is the natural logarithm of lag of per capita income (PCI), $$lnPCY_{st - 1}^{2}$$ denotes the squared natural logarithm of per capita income (PCI) that captures the non-linearities between income and flood fatalities. This implies $$\beta_{1} > 0 \;and\; \beta_{2} < 0$$, revealing an inverted U-shaped relationship between flood fatalities and $$ln{\text{PCY}}_{{{\text{it}} - 1}}$$. In Eq. (1) the turning point of PCI where flood fatalities (FF) are at a maximum, is given by: $$\frac{{\partial FF_{it} }}{{\partial lnPCI_{it - 1} }} = \beta_{1} + 2\beta_{2} lnPCI_{it - 1} = 0,{\text{turning point }}\left( {\uptau } \right) = {\text{exp}}\left( {\frac{{ - \beta_{1} }}{{2\beta_{2} }} } \right)$$. $$\left( {\frac{NCE}{{GSDP}}} \right)_{st - 1}$$ is the lag of the ratio of natural calamity expenditure (NCE) over GSDP and $$\left( {\frac{NCE}{{GSDP}}} \right)_{st - 1}^{2}$$ is the squared lag of the ratio of natural calamity expenditure (NCE) over GSDP. These two variables capture the non-linear relationship between natural calamity expenditure and flood fatalities. In Eq. (1), $$\beta_{3} > 0 \;and\; \beta_{4} < 0$$ reveal an inverted U-shaped relationship between flood fatalities and natural calamity expenditure (NCE). The turning point of natural calamity expenditure where flood fatalities (FF) are at the maximum is given by $$\frac{{\partial FF_{it} }}{{\partial \left( {\frac{NCE}{{GSDP}}} \right)_{st - 1} }} = \beta_{3} + 2\beta_{4} \left( {\frac{NCE}{{GSDP}}} \right)_{st - 1} = 0, {\text{turning point }}\left( {\uptau } \right) = \left( {\frac{{ - \beta_{3} }}{{2\beta_{4} }} } \right).$$

In Eq. (2), $\alpha_{1} > 0\; and \;\alpha_{2} < 0$ reveal an inverted U-shaped relationship between the population affected and PCI. The turning point of PCI at which the population affected is maximum is given by $\left( {\uptau } \right) = {\text{exp}}\left( {\frac{{ - \alpha_{1} }}{{2\alpha_{2} }} } \right)$. In addition, $\alpha_{3} > 0\; and\; \alpha_{4} < 0$ represent the inverted U-shaped relationship between the population affected and NCE. In other words, the turning point of NCE where the population affected are at the maximum, is given by $\left( {\uptau } \right) = \left( {\frac{{ - \alpha_{3} }}{{2\alpha_{4} }} } \right)$. In Eq. (3), $\delta_{1} > 0\; and\; \delta_{2} < 0$ show the inverted U-shaped relationship between flood damages and PCI. The turning point of PCI at which flood damages are at the maximum, is given by $\left( {\uptau } \right) = {\text{exp}}\left( {\frac{{ - \delta_{1} }}{{2\delta_{2} }} } \right)$. Moreover, $\delta_{3} > 0 and \delta_{4} < 0$, which reveals an inverted U-shaped relationship between flood damages and NCE. The turning point of NCE at which flood damages are at the maximum, is given by $\left( {\uptau } \right) = {\text{exp}}\left( {\frac{{ - \delta_{3} }}{{2\delta_{4} }} } \right)$.

${\text{SED}}_{{\text{st }}}$ is the state election dummy equal to 1 for the years in which elections are held in a State; otherwise, it is zero. ${\text{NED}}_{{\text{st }}} { }$ is the national election dummy, which is equal to 1 for the years in which elections are held at the national-level; otherwise, it is zero. ${\text{PAD}}_{{\text{st }}}$ takes on a value of 1 if the State and Centre have the same political party in power; otherwise, it is zero. The flood-affected area dummies (AD) are created by categorizing the state-wise flood-affected area into four categories. This helps control flood magnitude in the empirical analysis. For a detailed discussion, see the section on ‘Data sources.’

The variable of interest is economic development (proxied by the lag of PCI and squared lag of PCI). The other variable of interest is the lag of government responsiveness and the squared lag of government responsiveness (proxied by natural calamities expenditure as % of GSDP). The control variables $({\text{Z}}_{{{\text{st}} - 1{ }}} )$ are common to all three econometric specifications. These variables include proxies for coping strategies, such as financial development (measured as total credit outstanding as a percentage of GSDP), forest cover (measured as log of forest cover/total state area), and flood control and prevention expenditure measures. The other control variables include rural head count ratio: the number of rural people living below the poverty line (as a percentage of rural population), population density, the severity of floods, an election year, and political alignment. We have used the 1-year lagged values of the explanatory variables to control for potential endogeneity issues. $\theta_{s}$ is time-invariant state-specific fixed effects whereas $\gamma_{t}$ captures time-specific heterogeneity. $\mu_{st}$ is a normally distributed error term.

The outcome variables are flood fatalities and population affected in Eqs. (1) and (2), respectively. Flood fatalities and population affected are measured as the number of people who were killed and affected due to floods in different states in the respective years. Both the outcome variables are non-negative count variables. As shown in Appendix Table A.1, the variances of the two variables exceed their respective means implying that flood fatalities and population affected variables are overdispersed. This violates the normality assumption of the Ordinary Least Squares (OLS) model. In this particular case, the OLS method of estimation yields biased results. Therefore, using FE Poisson and Negative Binomial models is more appropriate to estimate Eqs. (1) and (2). In particular, the overdispersion of dependent variables violates the fundamental assumption of the FE Poisson model. Therefore, the Negative Binomial is favoured over the FE Poisson. The Deviance goodness-of-fit and Pearson goodness-of-fit test results suggest that FE Negative binomial is appropriate in our dataset. However, overdispersion test results suggest that the FE Poisson estimate is appropriate for our dataset (See Appendix Table A.2)^21^. Therefore, we have estimated both models.

However, as^9^ pointed out, a limitation of the negative binomial is that it controls less robustly for unobserved time-invariant country-specific heterogeneity. Furthermore, FE Negative Binomial efficiently controls the time-invariant unobserved fixed effects when the number of cross-sectional units is less than 20 (in our case, the number of states = 19)^22^. In contrast to the Negative Binomial, the FE Poisson completely controls the unobserved time-invariant fixed effects, which could be a plausible cause of overdispersion^9,23^. We estimate Eqs. (1) and (2) using both FE Negative Binomial and FE Poisson methods, keeping in mind the advantages and disadvantages of both models. A few studies used FE Negative Binomial and FE Poisson estimates in the disaster fatalities dataset^9,11^. We apply the FE Tobit estimation technique to estimate Eq. (3) as the outcome variable is flood damages $FD_{st }$ is defined as ln [ $\left( { \frac{Total \;Damages }{{GSDP}}} \right) + 1 ]$ has a lot of observations that have zero values.

### Data sources

In this study, we have compiled data for India’s 19 major states from 1980 to 2011. The state-wise flood information such as fatalities, the population affected, flood damages, and area affected by floods are taken from Central Water Commission (CWC)^24^ reports, Ministry of Jal Shakti, Department of Water Resources, River Development and Ganga Rejuvenation, Government of India. The CWC reports do not provide state-wise flood magnitude, one of the important measures to determine the severity of floods. Thus, we have followed the method described by^25^ for estimating the state-wise severity of floods. To measure the severity of a flood, we have divided the area affected by floods into four categories for 19 states from 1980 to 2011. The first quartile (the area affected is less than 25%) denoted by less severe flood dummy is set equal to 1; otherwise, zero; the second quartile (moderate flood), the state-wise area affected by floods lies between 25 and 49% is equal to 1; otherwise, zero; the third quartile (high flood), the area affected by floods lies between 50 and 74% is equal to 1; otherwise zero; the fourth quartile (severe flood), the area affected by floods lie above 75% is equal to 1; otherwise zero. For a detailed discussion regarding the definition of all categories of the flood magnitude, see Appendix Table A.1.

We have taken Gross State Domestic Product (GSDP) data at a constant price from the Ministry of Statistics and Program Implementation, Government of India. The natural calamity expenditure and expenditure for flood control are taken from the Reserve Bank of India. The forest cover data are taken from *Land Use Statistics.* The financial development (proxied by credit outstanding) has been taken from the Economic and Political Weekly (EPW) research foundation. The state-wise population data are available decennially from the Census of India. We have linearly interpolated the population data to fill up the gap year population. The rural poverty head count ratio data for each State is taken from the Planning Commission’s^26^ report.

We have included dummies for state elections, national elections, and political alignment in our econometric specification. State elections and political alignment between the centre and states influence the degree of flood fatalities, and healthy centre-state coordination is required to mitigate the effects of floods^11^. The election data are taken from the Election Commission of India. We have constructed the political alignment dummy using the Election Commission of India dataset. The definition and summary statistics of all variables are shown in Appendix Table A.1.

On average, 93 people were killed annually, and around 2 million people were affected due to floods in major 19 over the period 1980–2011. The average per capita income is Rs. 21,774 (712 US$), and the government spends around 0.233% of GSDP on natural calamities. Based on per capita income, as suggested by^10^, we have divided our dataset into high-income (rich) and low-income (poor) states. The low-income states include the north-east and east states of India. The 9 high-income states are Andhra Pradesh, Gujarat, Haryana, Himachal Pradesh, Karnataka, Kerala, Maharashtra, Punjab, and Tamil Nadu. The 10 poor states (including north-east and east states) include Assam, Bihar, Jammu and Kashmir, Madhya Pradesh, Manipur, Odisha, Rajasthan, Tripura, Utter Pradesh, West Bengal.

In the high-income states, on average, around 80 people were killed, and 1.06 million were affected due to floods over the period 1980–2011. Moreover, high-income states’ average per capita income is Rs. 27,794.76 (908.3 US$), and direct government spending for evacuation, relief distribution, and rehabilitation purposes is around 0.24% of GSDP. In the low-income states, 105 people were killed, and around 3 million people were affected annually. The average per capita income of low-income states is Rs.16354.6 (534.5 US$), and expenditure on natural calamities is around 0.23% of GSDP. The data shows that more people were killed, and many people were affected due to floods in low-income states than in high-income states. In addition, the high-income states are better-off due to their initial conditions of higher per capita income and expenditures on natural calamities than the low-income states.

## Results and discussion

### PCI and government responsiveness on flood fatalities

The regression results of FE negative binomial and FE Poisson estimates for Eq. (1) are reported in Table 1. The outcome variable is flood fatalities in all states. We use both regression models for estimation purposes because one model is advantageous over another. FE Poisson model controls more efficiently time-invariant unobserved state-specific heterogeneity. We introduce year dummy and state dummy to control time-variant and time-invariant unobserved state heterogeneity factors in all regression estimates. Also, we include cluster standard errors at the state level in all estimates.

**Table 1:** Flood fatalities, per capita income (PCI) and Government responsiveness (All States).

Variables	FE Poisson			FE negative binomial		
	*C*1	*C*2	*C*3	*C*4	*C*5	*C*6
${\text{lnPer capita income}}_{{{\text{t}} - 1}}$	7.337** (3.617)	9.239** (3.603)	7.403** (3.446)	7.404* (4.510)	6.583* (3.495)	9.888** (4.555)
${\text{lnPer capita incom}}_{t - 1}^{2}$	− 0.347** (0.173)	− 0.437** (0.172)	− 0.363** (0.170)	− 0.299 (0.212)	− 0.249* (0.151)	− 0.425** (0.209)
${\text{Government responsiveness}}_{{{\text{t}} - 1}}$	0.180 (0.729)	0.247 (0.752)	0.184 (0.831)	0.461 (0.499)	0.400 (0.512)	0.955* (0.568)
${\text{Government responsiveness}}_{t - 1}^{2}$	− 0.128 (0.306)	− 0.148 (0.323)	− 0.106 (0.335)	− 0.349* (0.188)	− 0.353* (0.197)	− 0.538** (0.227)
${\text{Financial development}}_{{{\text{t}} - 1}}$		− 0.016 (0.013)	− 0.023* (0.013)		− 0.014 (0.013)	− 0.015 (0.013)
${\text{Forest cover}}_{{{\text{t}} - 1}}$		0.016 (0.018)	0.019 (0.016)		− 0.002 (0.008)	− 0.006 (0.013)
${\text{Flood control Exp}}_{{{\text{t}} - 1}}$		− 0.065 (0.099)	0.075 (0.109)		− 0.110 (0.155)	0.082 (0.231)
State election		− 0.518*** (0.128)	− 0.592*** (0.134)		− 0.398*** (0.148)	− 0.587*** (0.161)
Political alignment		− 0.237* (0.137)	− 0.398*** (0.149)		− 0.037 (0.195)	− 0.408 (0.261)
National election		− 0.253 (0.427)	− 0.320 (0.406)		0.167 (0.440)	− 0.3840 (0.410)
Rural head count ratio		0.003 (0.012)	0.013 (0.011)		0.010 (0.016)	0.043** (0.020)
Moderate flood dummy			0.441 (0.478)			1.304*** (0.433)
High flood dummy			0.887*** (0.266)			1.697*** (0.337)
Severe flood dummy			1.468*** (0.262)			2.356*** (0.342)
Population density			0.0009 (0.002)			0.003 (0.002)
*Turning point:* Per capita income (in Rs)	(38,803) (1,268 US$) (90%ile)	(38,424) (1,256 US$) (90%ile)	(26,980) (882 US$) (75%ile)	(236,756) (7,737 US$) (100%ile)	(533,794) (17,444 US$) (100%ile)	(111,843) (3,655 US$) (100%ile)
*Turning point:* Government responsiveness (as % of GSDP)	0.70 (95%ile)	0.83 (97%ile)	0.87 (97%ile)	0.66 (94%ile)	0.57 (93%ile)	0.89 (97%ile)
log-likelihood	− 31,795	− 30,727	− 25,966	− 2767	− 2764	− 2710
No. of states	19	19	19	19	19	19
observations	589	589	589	589	589	589

Clustered standard errors at state-level are reported in parentheses. The level of significance at ***p < 0.01, **p < 0.05, *p < 0.1. The dependent variable is flood fatalities. Time-invariant state and year fixed effects are included in all models. The low flood is the baseline dummy variable.

The coefficient of per capita income (PCI) is positive, and its squared coefficient is negative and significant without taking into account control variables in FE Poisson estimation (see column C1 of Table 1). These results indicate a non-linear relationship between deaths from floods and per capita income, and it follows an inverted U-shaped relation with respect to per capita income. Our findings are mirror findings by^7,9^. The turning point estimate of PCI is Rs. 38,803 (1268 US$), which lies at the 90th percentile distribution of our data sample (see column C1). The results imply that deaths from floods increase when per capita income is less than the turning point of PCI at Rs. 38,803 (1268 US$), and deaths decline further when income rises beyond the turning point of PCI at Rs. 38,803 (1268 US$).

**Figure 1:**
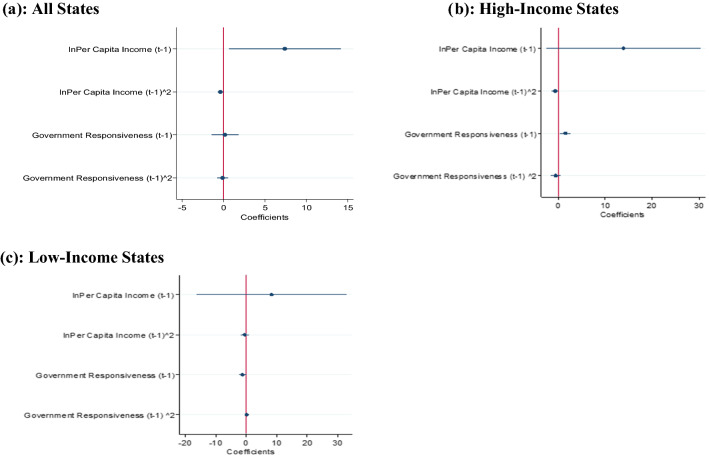
Coefficient plot—flood fatalities and income and Government responsiveness.

Still, we find the inverted U-shaped relationship between flood fatalities and PCI after considering other control variables such as disaster adaptation measures and political factors (see column C2 of Table 1). The turning point of PCI is 38,424 (1256 US$), which is lower than Rs. 38,803 (1268 US$). In our final regression estimates, the relation for PCI remains inverted U-shaped after controlling flood magnitude in column C3. The coefficient plots are shown in Fig. 1a–c, which explain the non-linear relationship between per capita income and flood deaths for All, high-income, and low-income states. Here, we provide the plot for the key variables used in the study. The coefficient of PCI is positive, and the coefficient of its squared term is negative, which shows that flood fatalities in all states are higher in the initial phase, and then deaths decline as PCI increases further.

**Figure 2:**
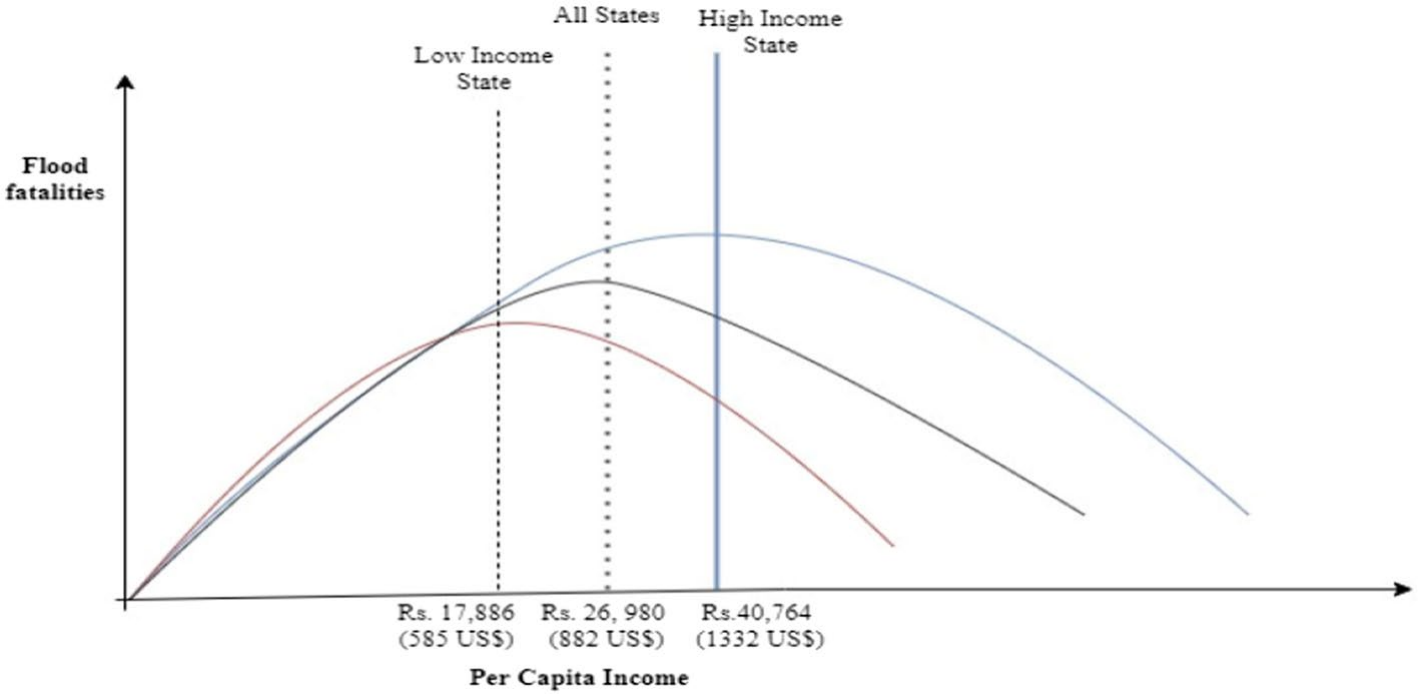
Turning point for flood fatalities with respect to per capita income-all states, high-income, and low-income states.

The turning points of per capita income are estimated using the linear and quadratic coefficients of the respective variables. Moreover, we use sample data to estimate the percentile value of the turning point of per capita income. In all states, the turning point of PCI is Rs. 26,980 (882 US$), which indicates that fatalities increase initially till the threshold income and thereafter decline with a further rise in PCI (see Fig. 2). We calculate the flood-related deaths using our sample data. The number of deaths from floods is 47,328 when the PCI is less than the turning point. When PCI crosses the turning point income, the flood fatalities reduce to 9408.

The magnitude of the turning point of PCI declines further from 1268 US$ to 882 US$ (lies at the 75th percentile distribution of our data) after controlling socio-economic and political covariates. Our estimates suggest that flood fatalities increase with increasing PCI up to 882 US$, then it declines when PCI is above 882 US$. We argue that we find the non-linear (inverted U-shaped) relationship in all three estimations (see columns C1, C2, and C3 of Table 1); however, the magnitude of the turning point of PCI varies.

We also estimate Eq. (1) using FE Poisson model to examine the non-linear (inverted U-shaped) relationship between per capita income and flood deaths for high-income and low-income states. The Poisson estimates are presented in Appendix Table A.3. To account for regional differences, we segregate our data into high-and low-income states. Higher flood fatalities and larger number of people are affected by floods in low-income states due to their higher poverty levels (measured by head count ratio) and inadequate disaster-resilient infrastructure (proxied by public expenditure on flood control and irrigation) (see Appendix Table A.1).

In the case of high-income states, we find that the coefficient of the linear term of per capita income is positive and statistically significant, and the coefficient of the squared term of income is negative and significant. This implies that the behavior of flood deaths is highly non-linear and follows the inverted U-pattern with respect to per capita income for high-income states (see Appendix Table A.3). This nonlinear relationship between flood deaths and income is presented in Fig. 1b. High-income states experience a lower level of flood deaths because higher income and stronger financial sectors help mitigate the effects of floods. Our estimates show a turning point of Rs. 40,764 (1332 US$), which suggests that flood deaths increase with a per capita income of less than Rs. 40,764 and then deaths decrease in per capita income for high-income states. Flood fatalities stood at 19,177 when the per capita income was less than the turning point income. As PCI crosses the turning point income, flood fatalities reduce to 3912 (see Fig. 2). Therefore, we find the existence of non-linearity (inverted U-shaped), as suggested by the theory.

For low-income states, the positive coefficient on per capita income and the negative coefficient on squared per capita income indicate the non-linear (inverted U-type) relationship between income and flood fatalities (see Appendix Table A.3). However, both coefficients are insignificant, implying that the non-linear relationship is weak. Our estimates show that deaths from floods are 25,025 when the PCI is less than the turning point income of Rs. 17,886 (585 US$). Furthermore, if PCI crosses the turning point income, flood fatalities reduce to 8622 (see Fig. 2). We find that low-income states experience higher deaths from floods than high-income states. This is because of the initial conditions of poverty and lack of adequate infrastructure in poor states.

Apart from income’s role in preventing flood fatalities, government responsiveness (proxied by government expenditure on natural calamities) plays a crucial role in mitigating flood fatalities (see Table 1). The coefficients of natural calamity expenditure are positive, and the squared terms are negative and insignificant. This implies an inverted U-shaped relationship between natural calamity expenditure and flood fatalities; however, this relationship is weak (see column C1 of Table 1). Furthermore, the relation remains inverted U-type with respect to natural calamity expenditure after considering other control variables (columns C2–C3 of Table 1). Moreover, it can be observed that the parametric estimates of the FE Poisson model are larger than their negative binomial estimates. The larger magnitudes of coefficients of variables in the FE Poisson estimate can be attributed to complete control for unobserved state-specific effects^9^. The coefficient plots are shown in Fig. 1a–c, which explain the non-linear relationship between government responsiveness and flood deaths for All, high-income, and low-income states. Flood fatalities increase with government responsiveness at low levels of governance and decrease with responsiveness at high levels of governance.

**Figure 3:**
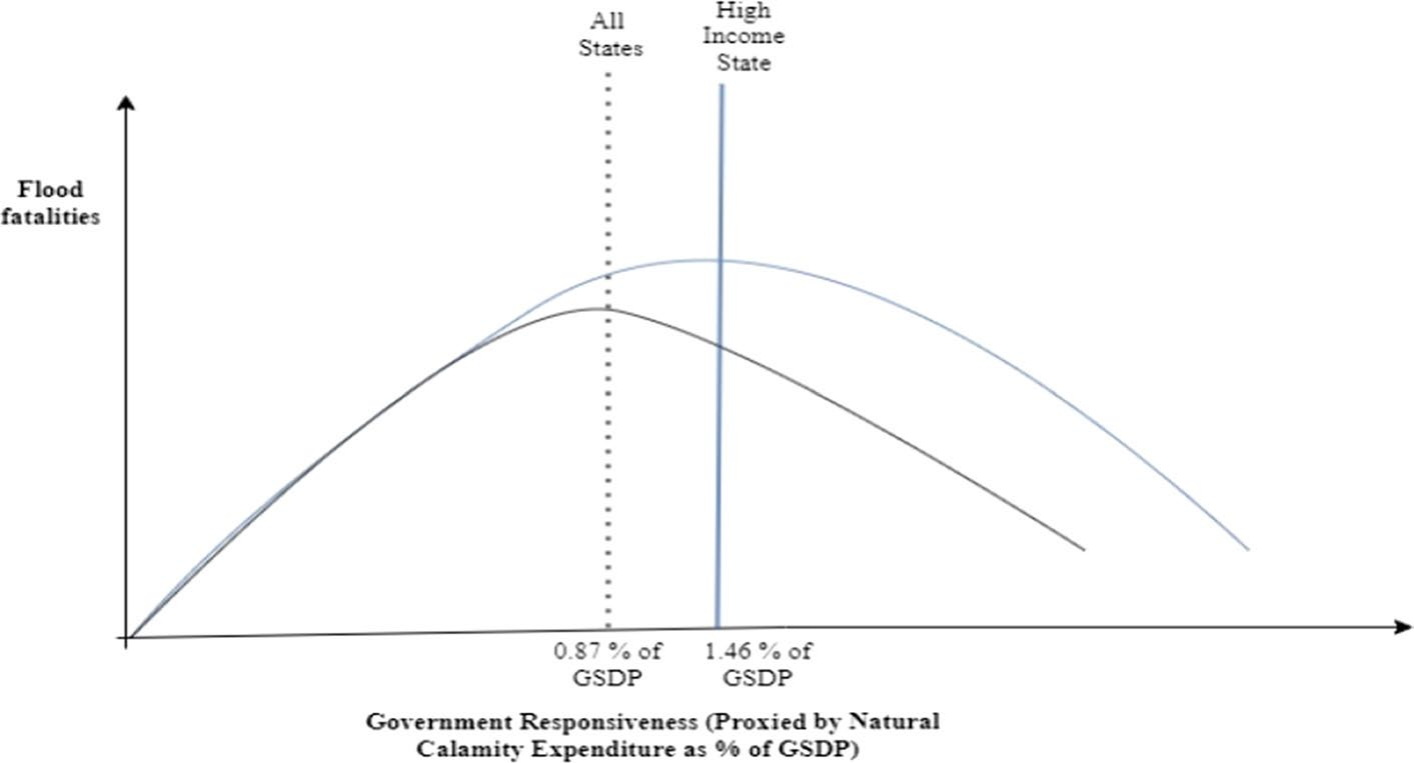
Turning point for flood fatalities with Government responsiveness—all states, high-income, and low-income states.

The turning point of the natural calamity expenditure as a percentage of GSDP is 0.87 (lies at 97% percentile, column C3 of Table 1). The estimates suggest that flood fatalities increase when natural calamity expenditure (NCE) as a percentage of GSDP is less than 0.87%, then it declines further when natural calamity expenditure as a percentage of GSDP is above 0.87%, but the relationship is weak. The inverted U-type relationship between NCE and deaths from floods is shown in Fig. 3. The results suggest that the current government expenditure for the mitigation of flood risk is not adequate. Around 53,200 people have been killed in all states due to floods when natural calamity expenditure as a percentage of GSDP is lower than 0.87% (turning point). Nearly 3536 people have died when the turning point of NCE as a percentage of GSDP reaches above 0.87%. Therefore, higher government spending to protect citizens against natural calamities is essential to reduce flood mortality.

In high-income states, flood fatalities are 22,883 when natural calamity expenditure as a percentage of GSDP is lower than 1.46% (turning point). Flood deaths reduce to 206 when natural calamity expenditure as a percentage of GSDP crosses the turning point. The results suggest that rich states with better spending on natural disasters suffer less death from floods. Greater government spending helps mitigate the effects of floods. However, low-income states with relatively lower ability to spend on disasters experience a U-shaped relationship between flood fatalities and government responsiveness. The governance responsiveness turning point lies outside our dataset, suggesting that government responsiveness provides protection but at a diminishing rate. This implies that states with lower income and inadequate government responsiveness are more likely to suffer greater flood-related deaths than developed states. This is consistent with^4^ findings, which suggest that countries with higher economic development are less likely to experience disaster deaths.

Additionally, disaster adaptation measures such as financial development, forest cover, and flood control expenditure are not sufficient to alleviate flood fatalities. The state election and existing political alignment between State and the Centre help to minimize flood fatalities. This finding is consistent with the results of^11^. The findings suggest that the central government releases favorable grants and soft loans to respective state governments with the same or coalition political party ruling the State. Moreover, if a flood disaster occurs in the state election year, incumbent state governments try to manage the flood calamity efficiently. The management work further helps the ruling government to enhance its prospects of winning the election. Also, the flood magnitude dummy (moderate, high, and severe) is positively related to flood fatalities (see column C3). This is in line with the findings of^9^ and^11^. The less developed states with a higher poverty ratio and higher population density are likely to experience higher flood fatalities.

### PCI and Population affected by floods

**Figure 4:**
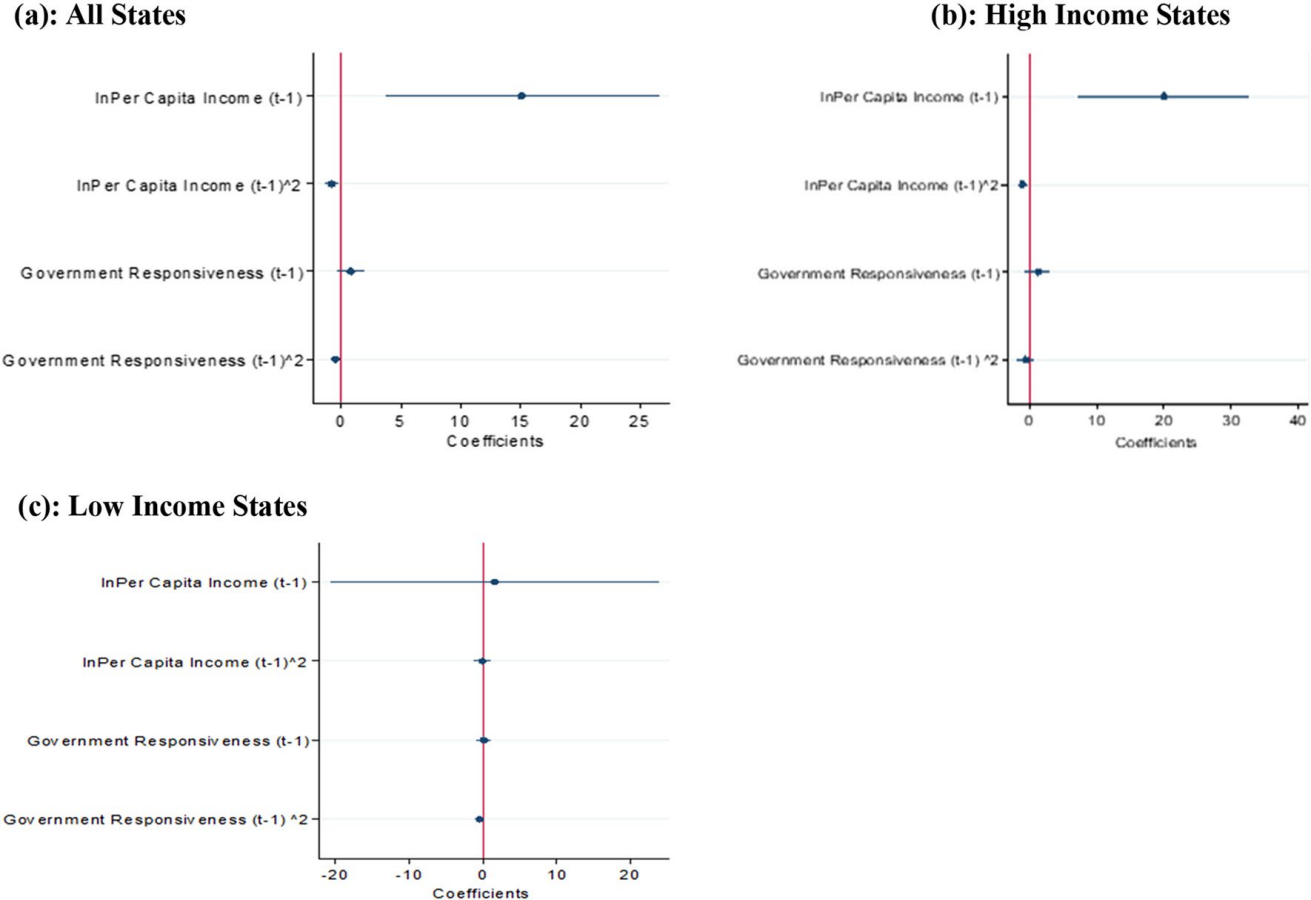
Coefficient plot—population affected and Income and Government responsiveness.

We evaluate the impact of PCI and government responsiveness on the population affected by floods, controlling for socio-political and flood magnitudes in high-and low-income states. The FE Poisson method is used to estimate Eq. (2), and the results are reported in Tables 3 and Appendix A.2. Our results show that an inverted U-type relationship between per capita income and the population affected by floods exists in all states, high-income, and low-income states. However, the inverted U-shaped relationship with respect to income is not significant for the low-income states. Therefore, a significant increase in PCI is required to mitigate the effects from floods in the poor states. The coefficient plots are shown in Fig. 4a–c, which shows the non-linear relationship between PCI and the population affected by floods in All, high-income, and low-income states.

**Figure 5:**
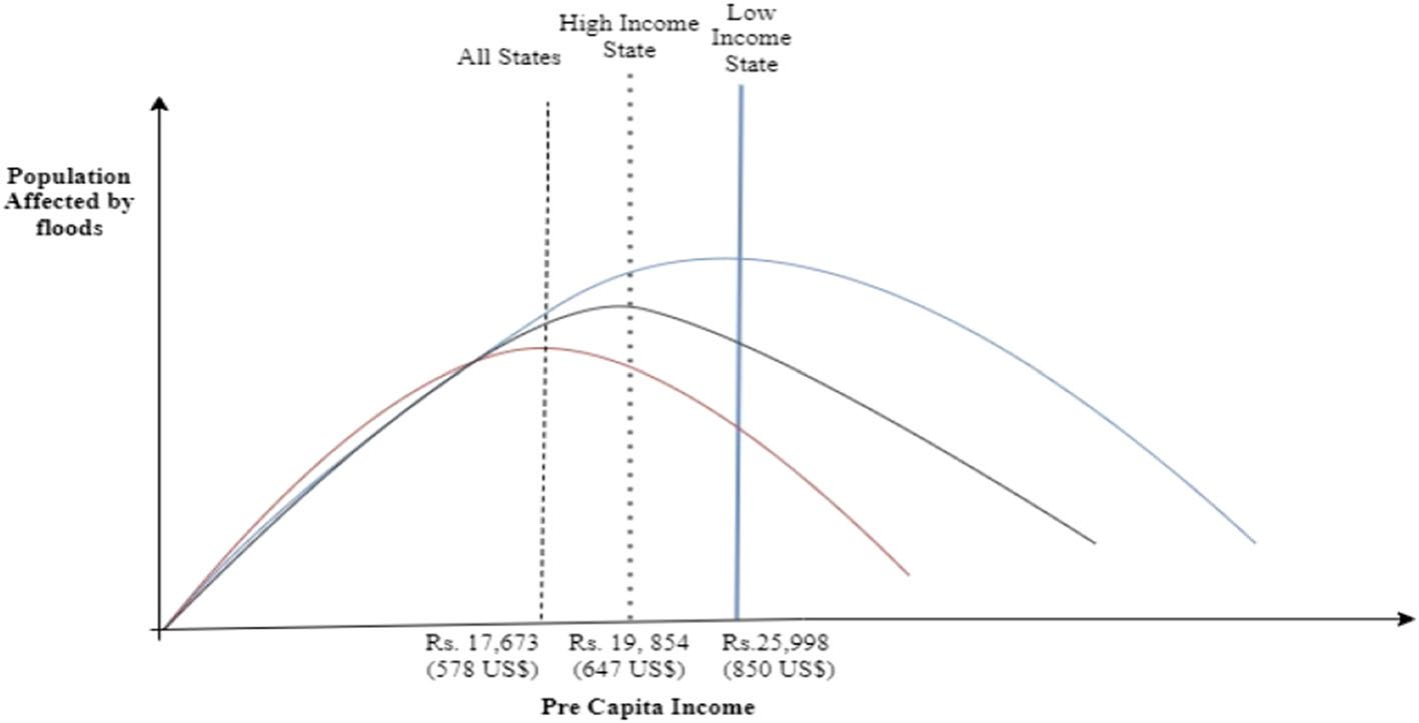
Turning point for population affected by floods with per capita income-all states, high income, and low-income states.

We estimate the turning point of PCI at which the population affected by floods is the maximum (see Fig. 5). The population affected was 855.9 million in all states when the PCI was less than the turning point PCI (578 US$). Thereafter, the population affected by floods declined by 343.7 million when income crossed the turning point PCI. The population affected in high-income states was 179.7 million when the PCI was less than the turning point PCI (647 US$). When income crossed the turning point PCI, the population affected reduced to 126.1 million. In low-income states, the population affected was 839.7 million when the PCI was less than the turning point PCI (850 US$). When income crossed the turning point PCI, the population affected reduced to 54.2 million. We conclude that poor states require higher turning point PCI to lessen the number of people affected by floods than rich states.

In addition, we employ the FE Poisson estimation method to examine the non-linear relationship between government responsiveness and the population affected by floods in All, high-income, and low-income states using Eq. (2). The results of Poisson estimates are shown in Table 2 and Appendix Table A.3, and the coefficient plot of the key variables is shown in Fig. 4a–c. The estimated results show an inverted U-shaped relationship between government responsiveness and the population affected by floods in high-income and low-income states; however, the inverted U-type relation is weak for both states. The result concludes that government responsiveness is still ineffective in mitigating the disastrous flood impact in Indian states.

**Table 2:** Population affected, per capita income (PCI) and Government responsiveness (All States).

Variables	FE poisson			FE negative binomial		
	*C*1	*C*2	*C*3	*C*4	*C*5	*C*6
${\text{lnPer capita income}}_{{{\text{t}} - 1}}$	7.810* (4.834)	11.057* (5.862)	8.837* (4.845)	12.918** (6.023)	12.384** (5.586)	15.109** (5.828)
${\text{lnPer capita incom}}_{t - 1}^{2}$	− 0.377 (0.250)	− 0.557* (0.306)	− 0.452* (0.261)	− 0.639** (0.314)	− 0.613** (0.292)	− 0.772*** (0.294)
${\text{Government responsiveness}}_{{{\text{t}} - 1}}$	0.004 (0.585)	− 0.043 (0.526)	− 0.024 (0.508)	0.406 (0.515)	0.419 (0.509)	0.817 (0.558)
${\text{Government responsiveness}}_{t - 1}^{2}$	− 0.219 (0.247)	− 0.188 (0.218)	− 0.061 (0.199)	− 0.396** (0.183)	− 0.425** (0.207)	− 0.453** (0.218)
${\text{Financial development}}_{{{\text{t}} - 1}}$		0.030 (0.026)	0.022 (0.019)		0.020 (0.021)	0.005 (0.017)
${\text{Forest cover}}_{{{\text{t}} - 1}}$		0.001 (0.021)	− 0.003 (0.011)		0.001 (0.014)	0.001 (0.013)
${\text{Flood control exp}}_{{{\text{t}} - 1}}$		0.006 (0.134)	0.174 (0.100)		0.048 (0.143)	0.246** (0.112)
State election		− 0.288* (0.163)	− 0.285** (0.138)		− 0.363* (0.188)	− 0.434*** (0.148)
Political alignment		0.182 (0.134)	0.126 (0.130)		0.128 (0.160)	− 0.052 (0.130)
National election		0.948 (0.959)	0.616 (0.842)		1.090 (1.057)	0.257 (0.874)
Rural head count ratio		− 0.015 (0.018)	− 0.007 (0.017)		0.015 (0.026)	0.024 (0.024)
Moderate flood dummy			0.956** (0.404)			0.946*** (0.238)
High flood dummy			1.227*** (0.370)			1.523*** (0.288)
Severe flood dummy			2.275*** (0.318)			2.589*** (0.253)
Population density			− 0.003 (0.002)			− 0.001 (0.003)
*Turning point:* Per capita income (in Rs)	30,929 (1011 US$) (83%ile)	20,512 (670 US$) (59%ile)	17,673 (578 US$) (50%ile)	24,267 (793 US$) (70%ile)	24,511 (801 US$) (70%ile)	17,760 (580 US$) (50%ile)
*Turning point:* Government responsiveness (as % of GSDP)	0.01 (2%ile)	–	–	0.51 (91%ile)	0.49 (91%ile)	0.90 (97%ile)
Log-likelihood	− 1089	− 1070	− 877	− 849	− 844	− 762
No. of states	19	19	19	19	19	19
observations	589	589	589	589	589	589

Clustered standard errors at state-level are reported in parentheses. The level of significance at ***p < 0.01, **p < 0.05, *p < 0.1. The dependent variable is the population affected by floods. Time-invariant state and year fixed effects are included in all models. The low flood is the baseline dummy variable.

**Figure 6:**
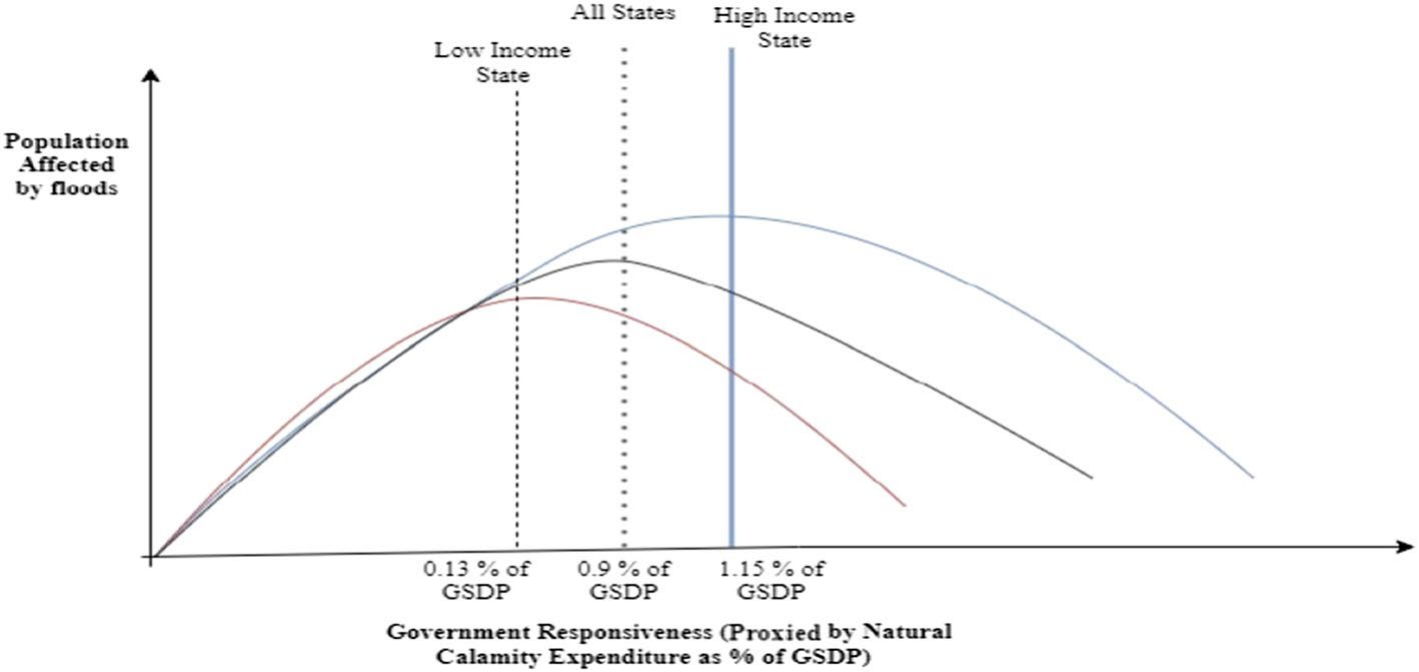
Turning point for population affected by floods with Government responsiveness—all states, high income, and low-income states.

We also estimate the turning point of government responsiveness and the number of people affected by floods below and above the turning point of government responsiveness (see Fig. 6). In all states, population affected was 1149.6 million when natural calamity expenditure as a percentage of GSDP was lower than 0.9% of GSDP (turning point). The number of persons affected reduced to 50.02 million when natural calamity expenditure as a percentage of GSDP crossed the turning point. In high-income states, the population affected was 278.6 million when natural calamity expenditure as a percentage of GSDP was lower than 1.15% of GSDP. If government responsiveness crossed the turning point, the number of persons affected reduced to 17.1 million. In low-income states, the population affected was 256 million when natural calamity expenditure as a percentage of GSDP was lower than 0.13% of GSDP. If government responsiveness crossed the turning point, the population affected reduced to 637.9 million.

Moreover, financial development, forest cover, and flood control expenditure have no significant effect on lowering the population affected by floods. On the other hand, the occurrences of state elections have significantly declined the flood impacts. Furthermore, the flood magnitude dummies remain significant in all Poisson specifications, implying that a larger population is affected due to severe flooding.

We estimate Eq. (2) using FE negative binomial for robust results, and results are presented in columns C4–C6 in Table 2. This exercise is done only for all Indian states. The inverted U-shaped relationship remains the same with respect to PCI. However, the turning point of income moderately varies with the FE Poisson estimate. In addition, non-linear relation is continued with respect to natural calamity expenditure throughout the models (see Table 2). Moreover, results show that severe floods adversely affect the population in Indian states. In sum, the FE negative binomial produces mirror results of FE Poisson estimates.

### PCI and government responsiveness on flood damages

We evaluate the effect of PCI and government responsiveness on flood damages, controlling socio-political and severity of floods. Using the FE Tobit model, we estimate Eq. (3), and the results are presented in Table 3. The outcome variable is flood damage over GSDP, which consists of many zeros, and the data is truncated from below. Therefore, it is appropriate to use Tobit estimation in our dataset. The flood damages include damage to crops, houses, and public infrastructure, all expressed in monetary values. We control year and state fixed effects, and standard errors are clustered at the state level in all regression models. The coefficient on the lag of PCI is positive and significant, and its squared term is negative and significant without considering control variables.

**Table 3:** Flood damages, per capita income, and natural calamity expenditure (All states).

Variables	FE tobit estimate		
	*C*1	*C*3	*C*3
$${\text{lnPer capita income}}_{{{\text{t}} - 1}}$$	0.122*** (0.031)	0.127*** (0.042)	0.117*** (0.041)
$${\text{lnPer capita incom}}_{t - 1}^{2}$$	− 0.005*** (0.001)	− 0.005** (0.002)	− 0.005** (0.002)
$${\text{Government responsiveness}}_{{{\text{t}} - 1}}$$	0.007 (0.009)	0.008 (0.008)	0.012 (0.008)
$${\text{Government responsiveness}}_{t - 1}^{2}$$	− 0.004 (0.003)	− 0.005* (0.002)	− 0.006** (0.003)
$${\text{Financial development}}_{{{\text{t}} - 1}}$$		− 0.00002 (0.0001)	0.00002 (0.0001)
$${\text{Forest cover}}_{{{\text{t}} - 1}}$$		0.00007 (0.00006)	− 0.0001** (0.00005)
$${\text{Flood control and irrigation exp}}_{{{\text{t}} - 1}}$$		0.002 (0.001)	0.002 (0.003)
State election		− 0.002 (0.002)	− 0.003* (0.001)
Political alignment		− 0.0007 (0.002)	− 0.003 (0.002)
National election		0.005 (0.008)	0.002 (0.007)
Rural head count ratio		0.0002 (0.0002)	0.0003 (0.0002)
Moderate flood dummy			0.014*** (0.003)
High flood dummy			0.019*** (0.003)
Severe flood dummy			0.031*** (0.004)
Population density			− 6.91e −06 (0.00002)
*Turning point:* Per capita income (in Rs)	187,203 (US$ 6118) (100%ile)	269,975 (US$ 8823) (100%ile)	119,372 (US$ 3901) (100%ile)
*Turning point:* Government responsiveness (as % of GSDP)	0.81 (95%ile)	0.85 (96%ile)	1.1 (98%ile)
Log-likelihood	894	897	983
No. of states	19	19	19
Observations	589	589	589

Clustered standard errors at state-level are reported in parentheses. The level of significance at ***p < 0.01, **p < 0.05, *p < 0.1. The dependent variable is $\ln \left( {\frac{Flood\; Damages}{{GSDP}} + 1} \right)$*.* Time-invariant state and year fixed effects are included in all models. The low flood is the baseline dummy variable.

The results confirm that an inverted U pattern with respect to PCI has been established (see column C1 of Table 3). The inverted U pattern is sustained with respect to PCI after considering economic, political, and flood severity variables (see columns C2–C3 of Table 3). The estimated turning point of PCI in all models exceeded the PCI's maximum value in our sample, implying that a higher PCI is needed to reduce flood damage. These results are consistent with the findings of^8^. They found an inverted U-shaped relationship between damages due to floods and per capita income using a cross-country dataset spanning 1980–2004. Besides PCI’s role in mitigating the flood damages, government responsiveness (measured by government expenditure on natural calamity) plays an essential role in mitigating flood damages in Indian states. The coefficient of natural calamity expenditure is positive, and its squared term is negative and significant, except for the model shown in column C1. The results follow an inverted-U pattern with respect to natural calamity expenditure throughout the models. We also estimate the non-linear relationship between PCI and flood damages in high and low-income states. The Tobit estimates show an inverted U-shaped relation between PCI and flood damages in high and low-income states. However, the relationship is weak for the low-income states. The coefficients plot of these results is shown in the Appendix Fig. A.1 (see Supplementary Information).

The turning point of natural calamity expenditure as a percentage of GSDP marginally increased from 0.81% in column C1 to 1.1% in column C3. This suggests that flood damages increase with a rise in natural calamity expenditure as a percentage of GSDP up to 1.1%; thereafter, it declines with increasing natural calamity expenditure. In addition, a higher flood magnitude yields greater flood damage. Controlling flood magnitude and total area under forest cover, we find that financial development and expenditure on flood control measures have no impact on flood damage. Furthermore, we find that the state election year and political alignment have little effect on reducing flood damages to some extent.

## Conclusion

The contribution of our work is that we focus on the effects of two important dimensions of development: per capita income and government responsiveness on flood fatalities, the population affected, and damages due to floods. We use state-level panel data of 19 Indian states over the period 1980–2011. To take into account the aspect of regional heterogeneity, our entire sample is divided into high-income (rich) and low-income (poor) states. We find evidence of a non-linear (inverted U-shaped) relationship between per capita income and flood impact (measured via flood fatalities, the population affected, and flood damages) in high-income states. In the initial phases, deaths from floods increase in income but then start decreasing once those states continue to grow as income crosses the turning point. This inverted U-shaped relationship between per capita income and flood-related deaths is weak for low-income states. A plausible reason is that the poor states are not equally capable of generating adequate revenues and hence find it difficult to mitigate the effects of disasters. As^11^ has pointed out that states with higher per capita income are relatively more capable of investing in flood-precautionary measures such as rehabilitation, evacuation, relief distribution, and disaster warning systems.

Apart from analyzing the role of per capita income, we examine the relationship between the role of government responsiveness (using natural calamity expenditure) and deaths and damages caused by floods in high-income and low-income states. Interestingly, the estimate of expenditure on natural calamity is positive, and its squared term is negative, implying that the inverted U-type relationship remains intact. These estimates are statistically insignificant, indicating that government responsiveness has a lesser impact on reducing flood fatalities and the population affected in Indian states. Other factors, such as financial development, forest cover, and flood control expenditures, have no significant impact on reducing flood fatalities and damages. The state election has significantly reduced flood fatalities and flood damages, while political alignment between both center and State has little effect on minimizing flood impact. Moreover, the high severity of floods has caused greater damage and more fatalities.

To sum up, our empirical results suggest that states with higher per capita income can spend more on flood control and mitigation measures and constructing flood-resilient infrastructure to minimize flood impact. Moreover, the current level of government responsiveness is not adequate to mitigate flood fatalities and damages in both high-income and low-income states. In particular, the low-income states need special government attention to reduce the adverse effects of floods. We argue that the government must spend more on rehabilitation, relief distribution, evacuation, and flood warning measures to minimize future flood risk. In addition, other measures, such as long-term flood management policy, increased government spending on flood control and irrigation, and allowing forest cover to grow can considerably reduce the flood impact in Indian states.

## Supplementary Information

Supplementary Information.

## Data Availability

The raw data of this study are being shared. For this, all authors have given their consents to share these materials.
