# Supplementary material for: Role of income and government responsiveness in reducing the death toll from floods in Indian states
Source: Sci Rep. 2022 Oct 10;12:16978. doi: 10.1038/s41598-022-21334-w (PMC9551081; doi:10.1038/s41598-022-21334-w)
Supplement: Supplementary file 1 — Supplementary Information. [file 41598_2022_21334_MOESM1_ESM.docx]

**Role of income and government responsiveness in reducing the death toll from floods in Indian states**

**Appendix A:**

| **All States** | | | | | |
| --- | --- | --- | --- | --- | --- |
| **Variables** | **Definition** | **Mean** | **Std.** | **Min** | **Max** |
| Flood Fatalities | Number of people killed due to floods | 93.32 | 181.09 | 0 | 1390 |
| Population Affected | Population affected due to floods (in million) | 1.97 | 3.92 | 0 | 30 |
| Flood Damages | Flood damages (as % of GSDP) | 0.73 | 1.56 | 0.0 | 15.13 |
| Per Capita Income (PCI)# | GSDP (in Rs) at 2004 prices/State-wise population | Rs. 21,774  (712 US$) | Rs. 12,279  (401 US$) | Rs. 4,483  (147 US$) | Rs.  69,786  (2281 US$) |
| Government Responsiveness (Proxy by Natural Calamity Expenditure) | Natural calamities expenditure by state government (as % of GSDP) | 0.233 | 0.274 | 0.0003 | 3.002 |
| Financial Development | Total credit outstanding (as % of GSDP at current price) | 20.01 | 14.08 | 1.91 | 95.02 |
| Forest Cover | Forest cover (as % of total state area) | 21.88 | 16.39 | 0.87 | 78.12 |
| Flood Control and Irrigation Expenditure | Flood control and irrigation expenditure (as % of GSDP) | 1.28 | 0.96 | 0.06 | 7.41 |
| State Election | Election held in the respective state in the respective year is equal to 1, otherwise zero | 0.22 | 0.41 | 0 | 1 |
| Political Alignment | State and Centre have the same political party in power is equal to 1, otherwise zero | 0.30 | 0.46 | 0 | 1 |
| National Election | National election year is equal to 1, otherwise zero | 0.28 | 0.45 | 0 | 1 |
| Rural Head count Ratio | Number of poor (as % of rural population) | 31.19 | 14.60 | 4.08 | 68.86 |
| Low flood Dummy | Area affected by floods is less than 25 percent is equal to 1, otherwise zero | 0.39 | 0.49 | 0 | 1 |
| Moderate Flood Dummy | Area affected by floods is lies between 25 to 49 percent is equal to 1, otherwise zero | 0.11 | 0.32 | 0 | 1 |
| High Flood Dummy | Affected by floods is lies between 50 to 74 percent is equal to 1, otherwise zero | 0.25 | 0.43 | 0 | 1 |
| Severe Flood Dummy | Area affected by floods is lie above 75 percent is equal to 1, otherwise zero | 0.25 | 0.43 | 0 | 1 |
| Population Density | Persons per square km | 361 | 254 | 26 | 1105 |
| **High-Income States $** | | | | | |
| Flood Fatalities | Number of people killed due to floods | 80 | 120 | 0 | 917 |
| Population Affected | Population affected due to floods (in million) | 1.06 | 2.47 | 0 | 18 |
| Per Capita Income (PCI)# | GSDP (in Rs) at 2004 prices/State-wise population | 27794.76  (908.3 US$) | 14003.47  (457.6 US$) | 6421.40  (209.8 US$) | 69786.98  (2280.6 US$) |
| Government Responsiveness (Proxy by Natural Calamity Expenditure) | Natural calamities expenditure by state government (as % of GSDP) | 0.24 | 0.27 | .00034 | 1.88 |
| Rural Head count Ratio | Number of poor (as % of rural population) | 23 | 11 | 6.4 | 56 |
| Flood Control and Irrigation Expenditure | Flood control and irrigation expenditure (as % of GSDP) | 1.35 | 1.1 | 0.15 | 7.41 |
| **Low-Income States (Including Northeast States) *** | | | | | |
| Flood Fatalities | Number of people killed due to floods | 105 | 222 | 0 | 1390 |
| Population Affected | Population affected due to floods (in million) | 2.79 | 4.73 | 0 | 30.35 |
| Per Capita Income (PCI)# | GSDP (in Rs) at 2004 prices/State-wise population | Rs.16354.6  (534.5 US$) | Rs.6949.9  (227.1 US$) | Rs.4482.5  (146.5 US$) | Rs.42560.3  (1390.9 US$) |
| Government Responsiveness (Proxy by Natural Calamity Expenditure) | Natural calamities expenditure by state government (as % of GSDP) | 0.23 | 0.28 | 0.0009 | 3.00 |
| Rural Head count Ratio | Number of poor (as % of rural population) | 39 | 13 | 4.08 | 69 |
| Flood Control and Irrigation Expenditure | Flood control and irrigation expenditure (as % of GSDP) | 1.21 | 0.8 | 0.06 | 5.05 |
| **Note:** # We have converted the Indian rupee into US$ after taking the average US$ exchange rate from 1980 to 2011. For example, the average exchange rate from 1980 to 2011 is 30.6 US$, and the average PCI is Rs. 21,774. We divide Rs. 21,774 by 30.6 US$ to get 712 US$ (value of the PCI in US$). **$- High-Income States -** Andhra Pradesh, Gujarat, Haryana, Himachal Pradesh, Karnataka, Kerala, Maharashtra, Punjab, Tamil Nadu. *****-**Low-Income States (Including Northeast States)-** Assam, Bihar, Jammu and Kashmir, Madhya Pradesh, Manipur, Odisha, Rajasthan, Tripura, Utter Pradesh, West Bengal. | | | | | |
| **Table A.1: Summary of the variables** | | | | | |

| **Test** | **Table 2 (C6)** | **Table 3 (C6)** |
| --- | --- | --- |
| (Cameron and Trivedi, 2009: 575; Overdispersion test) | *t*= 5.77  p-value= 0.000 | *t*= 2.88  p-value= 0.004 |
| Deviance goodness-of-fit | χ2= 49363.97  p-value=0.000 | χ2= 946.538  p-value=0.000 |
| Pearson goodness-of-fit | χ2= 61796.99  p-value= 0.000 | χ2= 1385.5  p-value= 0.000 |
| **Table A.2: Specification tests of FE Poisson (All States)** | | |

| **Variables** | **High Income States** | |  | **Low Income States (Including Northeast States)** | |
| --- | --- | --- | --- | --- | --- |
|  | **FE Poisson** | |  | **FE Poisson** | |
|  | **Flood fatalities** | **Population affected by floods** |  | **Flood fatalities** | **Population affected by floods** |
|  | *C1* | *C2* |  | *C3* | *C4* |
| ${lnPer Capita Income}_{t-1}$ | 13.954*  (8.358) | 20.030***  (6.478) |  | 8.372  (12.489) | 1.596  (11.347) |
| ${lnPer Capita Incom}_{t-1}^{2}$ | -0.657*  (0.360) | -1.012**  (0.343) |  | -0.427  (0.690) | -0.078  (0.579) |
| ${Government Responsiveness}_{t-1}$ | 1.551***  (0.581) | 1.253  (0.875) |  | -1.141*  (0.670) | 0.108  (0.500) |
| ${Government Responsiveness}_{t-1}^{2}$ | -0.532  (0.533) | -0.545  (0.576) |  | 0.176  (0.310) | -0.404  (0.291) |
| **Control variables@** | Yes | Yes |  | Yes | Yes |
| ***Turning point:*** Per Capita Income (in Rs) | (40,764)  (1,332 US$)  (82%ile) | (19,854)  (647 US$)  (36%ile) |  | (17,886)  (585 US$)  (65%ile) | (25,998)  (850 US$)  (91%ile) |
| ***Turning point:*** Government Responsiveness (as % of GSDP) | 1.46  (100%ile) | 1.15  (97%ile) |  | **3.24#**  (100%ile) | 0.13  (40%ile) |
| Log-likelihood | -9679 | -309 |  | -11015 | -476 |
| No. of States | 9 | 9 |  | 10 | 10 |
| Observations | 279 | 279 |  | 310 | 310 |
| Note: Clustered standard errors at state-level are reported in parentheses. The level of significance at *** p<0.01, ** p<0.05, * p<0.1. Time-invariant state and year fixed effects are included in all models. The low flood is the baseline dummy variable. **High Income States $-**Andhra Pradesh, Gujarat, Haryana, Himachal Pradesh, Karnataka, Kerala, Maharashtra, Punjab, Tamil Nadu. **Low Income States (Including Northeast States) &-**Assam, Bihar, Jammu and Kashmir, Madhya Pradesh, Manipur, Odisha, Rajasthan, Tripura, Utter Pradesh, West Bengal. **Control variables@-** Area affected by floods (as a percentage of state geographical area), Financial Development, Forest Cover, Flood Control and Irrigation Expenditure, State Election, Political Alignment National Election, Rural Head count Ratio. #-There exists a U-Shape relationship between flood fatalities and government responsiveness for low-income states. | | | | | |
| **Table A.3: Effects of Per Capita Income (PCI) and Government Responsiveness on Flood Fatalities and Population affected in High Income and Low-Income States** | | | | | |

**(7a): All States (7b): High-Income States**

**(7c): Low-Income States**

**Figure A.1: Coefficient plot - Flood Damages, Income and Government responsiveness**
